# Opt-Out Screening for Blood-Borne Viruses in Trauma and Orthopaedic Admissions From the Emergency Department: Are We Meeting the Standards?

**DOI:** 10.7759/cureus.98207

**Published:** 2025-11-30

**Authors:** Aaisha Shahbaz, Matthew Walker, Fahad Imami, Uday Mahajan, Rohma Shahbaz, Deepa Bose

**Affiliations:** 1 Trauma and Orthopedic Surgery, University Hospitals Birmingham, Birmingham, GBR; 2 General Surgery, Pakistan Air Force Hospital, Islamabad, PAK; 3 General Surgery, Combined Military Hospital Multan, Multan, PAK

**Keywords:** blood-borne viruses, clinical audit, emergency department, screening program, trauma and orthopaedic

## Abstract

Background

In the United Kingdom, blood-borne viruses (BBVs) such as Hepatitis B, Hepatitis C, and HIV continue to pose a significant health risk to the public. The national guidelines currently recommend that everyone attending the Emergency Department (ED) and having routine blood tests done is automatically offered testing for BBV, unless they choose to opt out. During their management, patients under the care of Trauma and Orthopaedics (T&O) are admitted from several different ED areas, which makes them a key group for analysing how consistently screening recommendations are adhered to.

Objectives

The aim of this study was to evaluate the efficiency of the national BBV opt-out screening guidelines' implementation for patients admitted to the T&O service from the ED and to analyse whether there are differences in screening practices across the various ED areas.

Methods

A retrospective clinical audit at Queen Elizabeth Hospital Birmingham was conducted, reviewing all T&O patients admitted from the ED between the 1^st^ of February and the 15^th^ of March 2025. Patients who were already screened within the past 12 months were not re-tested but were still recorded as compliant with screening guidelines as per hospital policy. The data were extracted from electronic clinical records and laboratory systems. Descriptive statistics and chi-square tests were then used to assess overall screening compliance and to compare screening rates across different ED areas (Resuscitation, Majors, and Minors/Others).

Results

Among the 89 acceptable admissions, the mean age was 59 ± 22 years. Overall, 53 (59.6%) patients had been tested for BBVs at the time of admission, while another 14 (15.7%) had a valid test from the past year. This brought total compliance with the screening guidelines to 67 (75.3%). The remaining 24.7% were neither screened nor had reasons documented for opting out. Six patients (6.7%) had tested positive for Hepatitis C, including three who were newly diagnosed. Screening rates varied across ED areas. Resuscitation had the highest compliance, with 24 (88.9%) patients screened, followed by Majors (33, 71.3%) and Minors/Other (10, 62.5%). These differences, however, did not reach statistical significance (p = 0.111).

Conclusion

Even though the majority of T&O admissions received BBV screening, almost one in four eligible patients were not tested, which represents a missed opportunity for early detection and prevention. Higher screening rates in the Resuscitation Unit suggest that automated testing pathways can improve compliance. Introducing BBV tests into the routine ED blood panels, along with increasing staff awareness, and ensuring proper documentation and record when patients opt out, could assist in reducing the gaps.

## Introduction

Blood-borne viruses (BBVs), including human immunodeficiency virus (HIV), hepatitis B (HBV), and hepatitis C (HCV), represent a significant public health concern in the United Kingdom [1,2]. Early diagnosis is crucial to enable prompt treatment, prevent onward transmission, and reduce healthcare costs associated with advanced disease [3]. Although effective antiviral medications are available, a large portion of those with BBVs remain undiagnosed [4-6]. This phenomenon constrains progress in reaching national elimination milestones for BBVs.

In April 2022, NHS England launched a funded programme for opt-out BBV testing in emergency departments (EDs) located in areas of high diagnosed HIV prevalence [7-9]. This initiative aims to normalise testing, reduce stigma, and identify undiagnosed infections among patients attending for unrelated medical or surgical issues, as improving BBV screening uptake is central to national elimination strategies for HIV and HCV, and EDs represent a major opportunity for case finding. The World Health Organization (WHO) has similarly emphasised BBV elimination, targeting an 80% reduction in new infections and a 65% reduction in related mortality by 2030 [10,11]. According to this initiative, any patient who presents to the ED and has blood tests performed as part of their management is automatically screened for BBVs unless they opt out of the programme, as this opportunistic opt-out screening method increases uptake across BBVs [12,13]. Public awareness posters are signposted in various areas of the ED for information and guidance.

As our hospital is a major trauma centre, we receive a high volume of Trauma and Orthopaedic (T&O) patients who present to almost all areas of our ED, i.e. Resuscitation, Majors and Minors/Others. Therefore, this cohort is an important group for assessing the uniformity of BBV screening implementation across different areas of the ED and for identifying areas for further intervention to address inequalities [14].

The aim of this audit was to determine compliance with BBV screening standards among patients admitted to the T&O department from the ED and to identify the ED location where screening practices did not meet required standards.

## Materials and methods

This was a single-cycle clinical audit in which compliance with the opt-out BBV screening standard was assessed among patients admitted to the T&O department from the ED at Queen Elizabeth Hospital. The total duration of this audit cycle was from February 1st to April 30th, 2025, which allowed sufficient time for data collection, analysis, and presentation of findings and recommendations. Data collection for this data cycle lasted from 1st February to 15th March 2025. The audit employed a retrospective observational design. All the patients admitted to T&O from the ED from 1st February to 15th March 2025 were included in this study, regardless of gender or presenting complaints. According to hospital policy, if a patient had a BBV test within the last 12 months, their samples were not repeated. Patients with BBV testing done in the previous 12 months were considered compliant with the screening standard. Patients younger than 16 years of age were excluded according to national guidelines. No exclusions were made based on the diagnosis or treatment plan.

Data were obtained from the electronic patient records and laboratory results. The microbiology and virology results of each patient were reviewed to assess whether the BBV screening samples were sent to the laboratory on the date of admission from the ED. The results of those tests were also included in this study. To ensure data accuracy, a second reviewer independently verified 50% of extracted records. Other data fields included the admission date, the patient's age at admission, and the ED location where the admission occurred. The locations in our ED include the Resuscitation Unit, the Majors Unit, the Minors Unit and others, including Same Day Emergency Care (SDEC) or ambulatory care. The audit standard was set according to the national guidelines of 100% compliance with the hospital's opt-out BBV screening programme.

The data analysis was conducted using IBM SPSS Statistics for Windows, Version 25 (Released 2017; IBM Corp., Armonk, New York). Quantitative variables, i.e., patient age, were reported as mean ± standard deviation when the data were normally distributed, as assessed by the Shapiro-Wilk test. The primary outcome variable, compliance with opt-out BBV screening, was treated as a categorical variable and presented as frequencies and percentages. Comparisons between the categorical variables, including compliance across ED areas (i.e., Resuscitation, Majors, and Minors/Others), were performed using the chi-square test. A p-value of <0.05 was considered statistically significant.

This project was registered with the local clinical audit department under the CARMS Audit Code 23179. As it was conducted as part of a service evaluation, formal ethical approval was not required. Patient confidentiality was maintained throughout, and no identifiable data were collected.

## Results

A total of 89 patients were admitted from various areas of the ED to T&O during the data collection period from 1st February to 15th March 2025. The mean age of the cohort was 59 ± 22 years (mean ± SD). Out of these 89 patients, 27 (30.3%) were admitted from the Resuscitation Unit, 46 (51.7%) were admitted from the Majors, and 16 (18%) were admitted from the Minors and Others. A total of 77 (86.5%) patients had their routine blood tests done in the ED, and 12 (13.5%) did not have any blood samples sent in the ED before they were admitted to the T&O department. No incomplete or missing laboratory or clinical records were identified for the included patients. If a BBV screening result was not present, the patient was classified as not screened.

Of these 89 patients, 53 (59.6%) had their BBV testing samples sent, 22 (24.7%) were not screened for BBV, and there was no documentation of reasons for opting out of the screening programme. A total of 14 (15.7%) patients had their BBV screening within the last 12 months; therefore, they were not eligible for screening during this admission. Out of the total of 67 (75.3%) patients whose screening was compliant with our standards, six (6.7%) patients were found to be positive for HCV total antibodies. Of these six patients, three were previously known to be positive and under treatment, and three were newly diagnosed.

In our analysis of BBV screening across different areas of the ED, we found that 13 (28.3%) patients admitted from Majors, out of a total of 46 admissions from this area, did not have their BBV screening opt-out completed. A total of 27 patients were admitted from the Resuscitation unit, and three (11.1%) patients did not have their samples sent for screening. Sixteen patients were admitted from Minors and Others, and among them, six (37.5%) did not have their screening performed. No statistical difference was found among these areas in their opt-out BBV screening compliance; i.e., p-value = 0.111 according to the chi-square test. The results are summarised in Table 1.

**Table 1:** Compliance with opt-out BBV screening standards by emergency department location

ED Location	BBV Screening Done n (%)	BBV Screening Not Done n (%)	BBV Screening Done in the Last 12 Months n (%)
Resuscitation Unit	23 (85.19%)	3 (11.11%)	1 (3.70%)
Majors	23 (50.00%)	13 (28.26%)	10 (21.74%)
Minors and Others	7 (43.75%)	6 (37.50%)	3 (18.75%)
Total	53 (59.55%)	22 (24.72%)	14 (15.73%)

## Discussion

This audit assessed adherence with the NHS England opt-out BBV screening programme among T&O admissions from the ED at Queen Elizabeth Hospital Birmingham between 1st February and 30th April 2025 [8,10]. The results show that 24.7% of eligible patients did not get assessed for BBV, even though national guidelines say that all individuals who undergo regular blood tests in EDs should automatically be offered HIV, HBV, and HCV testing unless they opt out.

We further found that the Resuscitation unit had the highest compliance rate, with only 11.1% of patients not receiving BBV screening. This is because all T&O admissions from Resuscitation arrive as Trauma Alerts. As part of our hospital's primary survey and assessment policy, the Major Trauma Blood Test Panel is ordered, which includes BBV screening tests automatically. This shows that automated system prompts decrease the chance of missing screening samples even in a high-pressure setting [15].

The NHS England ED opt-out screening initiative, launched in April 2022, aims to increase testing, reduce stigma, and detect undiagnosed illnesses. Interim national data from the five sites selected for deep dive analysis showed that, among patients having blood tests, approximately 49% were screened for HIV and around 46% for HBV and HCV, though uptake showed significant variability among sites [10,16]. The screening rate observed in this audit (75.3%) compares favourably with several national averages, suggesting a good but incomplete implementation locally. However, achieving full compliance remains essential to fulfilling England's public health objectives for the eradication of BBVs.

The 24.7% of patients who were not screened reflect a mix of system-level and operational barriers. Factors reported nationally include variability in how opt-out screening is rooted in electronic requesting systems, inconsistent staff awareness, and problems in integrating BBV tests into regular blood panels. Manual ordering or lack of automated prompts has been associated with poor uptake. Local workflow issues, such as high ED workload and the surgical admission process, may also bring about missed opportunities. Including BBV screening in the default blood order sets for all ED admissions could further enhance compliance [15].

Importantly, the discovery of reactive test results in this audit underscores the significance of BBV screening. National ED screening data demonstrated that new diagnoses are found across all demographic groups, including those without classical risk factors [16]. Routine opt-out testing, therefore, plays a crucial role in reducing stigma and ensuring fair access to early diagnosis and treatment.

From a public health standpoint, achieving near-universal BBV screening aids national elimination strategies. The UK aims to eradicate HCV as a public health threat by 2025 and HIV transmission by 2030 [8]. Missed screening opportunities delay diagnosis, hinder access to care, and risk ongoing transmission. Even when false-positive or resolved cases are detected, reassurance is provided to patients, and the opportunity for education promotes prevention and awareness.

This audit had several strengths. It evaluated the implementation of the NHS England opt-out BBV screening programme in a real clinical setting, focusing on a clearly defined T&O admission pathway. All eligible T&O patients were included, which reduced selection bias. The screening status was confirmed using objective laboratory results, which increased the accuracy of our findings. We incorporated BBV results from the last 12 months and ensured that patients whose samples were not repeated due to prior screening and testing were classified as compliant in accordance with our hospital policy. This audit further compared the screening practices across various locations in the ED, which helped in identifying variations in screening practices and highlighted areas that needed further intervention to increase compliance rates. Statistical analysis was also conducted to assess differences in compliance across distinct ED locations.

There are also certain limitations to this audit which need to be considered while interpreting the findings. As this is a retrospective review, the audit relies on the accuracy and completeness of electronic patient records and laboratory information, which may contain missing or inaccessible results. This audit could not determine why screening was not performed in patients who had no documentary evidence of opting out of the screening programme. Multiple reasons could have potentially contributed to the low level of compliance, including omission due to workflow, clinical acuteness, or staff factors. This audit was based on a single centre and only a single speciality pathway, i.e., T&O admissions were included, which can limit generalisability. Differences in screening rates in the ED were not statistically significant, which may reflect limited statistical power due to uneven distribution of admissions across ED areas. The lack of statistical significance (p = 0.111) may reflect limited power due to a small sample size and an uneven distribution of admissions across ED locations, rather than true equivalence in screening performance. Finally, as a single-cycle audit, it provides only a snapshot of current practice, and further repeat measurements need to be made after recommended interventions.

Based on the findings from this audit, the recommendations to our department were to include BBV screening in the standard ED blood panels, to ensure it is automatically included in routine bloods. Targeted staff education needs to be performed to reinforce awareness of the national screening requirements. Informing the inpatient admissions team to ensure that if any patient has missed the opportunity to be screened, the offer to send screening blood will be made to that patient whenever the next blood samples are due to be taken. Additionally, a future cycle of this audit is needed to ensure that screening practices meet national standards after interventions.

## Conclusions

This audit highlights that while most T&O admissions from ED received opt-out screening, almost a quarter of patients were not evaluated. This represents a missed opportunity for early diagnosis and prevention. Compliance with the screening programme was inconsistent across various ED areas, with automated pathways in the Resuscitation Unit achieving higher uptake, highlighting the importance of system-based prompts. Integrating BBV testing into standard blood panels, enhancing staff awareness, and improving documentation of patient opt-outs are recommended to achieve full adherence to national screening standards.
